# Clinical Progress on Management of Pneumonia Due to COVID-19 With Chinese Traditional Patent Medicines

**DOI:** 10.3389/fphar.2021.655063

**Published:** 2021-09-02

**Authors:** Ying Wu, Ping Zhong

**Affiliations:** Department of Neurology, Shanghai Shidong Hospital of Yangpu District, Shanghai, China

**Keywords:** COVID-19, Chinese patent medicines, clinical studies, traditional Chinese medicine, pharmacological evaluation

## Abstract

**Background:** The outbreak of new coronavirus has tremendously threatened the public health system worldwide, including China. Chinese patent medicines (CPMs) have greatly contributed to the prevention and treatment of this viral infection, as well as the recovery of patients with COVID-19 infection. Therefore, numerous experts and guidelines recommend to take CPMs to treat pneumonia due to COVID-19.

**Aim of the Study:** The present study reviewed CPMs recommended by the < Guidelines for diagnosis and management of COVID-19 (8^th^ edition)> regarding evidence of their efficacy from clinical studies and the underlying mechanisms, which will lay the foundation for clinical use of these CPMs for COVID-19.

**Methods:** The composition, efficacy, indications, history of use, and relevant clinical research on 14 recommended CPMs, including Huoxiangzhengqi capsules (pills, liquid, oral solution), Jinhuaqinggan granules, Lianhuaqingwen capsules (granules), Shufengjiedu capsules, Xiyanping injections, Xuebijing injections, Reduning injections, Tanreqing injections, Xingnaojing injections, Shenfu injections, Shengmai injections, Angongniuhuang pills, Suhexiang pills, were searched in both Chinese and English databases based on differences in stages of the disease and manifestations of such patients. Advantages of these CPMs over conventional treatments and their underlying mechanisms were explored by analyzing results from published articles and undergoing clinical trials.

**Results:** Findings from clinical studies and Chinese experience in using these CPMs showed that CPMs, when used in combination with conventional treatments, were effective in managing COVID-19 with few side effects.

**Conclusion:** CPMs have excellent efficacy in managing COVID-19 with a great potential for clinical use.

## Introduction

COVID-19 broke out in Wuhan, Hubei Province of China and spread to other regions in China since December 2019. In the following months, it has spread to other countries and become a global public health threat (The Lancet, 2020). It was nearly under control in China after taking proactive measures by the Chinese government and relevant authorities in the first few months. Chinese traditional patent medicines (CPMs) have been widely used based on the diagnosis according to the Traditional Chinese Medicine (TCM) theory and they showed superiority to general treatments. This reflects the advantages of TCM in fighting against COVID-19. TCM is not only helpful for patients with different symptoms but also for those at different stages of the disease. It is recommended to halt or slow the progression of this disease in either the observation period or when symptoms manifest (Chen et al., 2020b; Shao et al., 2020). An official report has shown that 91.5% of patients with COVID-19 have used TCM therapies in China and the efficacy is over 90% (http://www.gov.cn/). The present study aimed to review relevant clinical studies, underlying mechanisms, and clinical efficacy of 14 CPMs recommended by National Health Commission of People’s Republic of China in < Guidelines for diagnosis and management of COVID-19 (8^th^ edition)>. This will facilitate the active application of these CPMs to patients with COVID-19 in other countries with an aim to control this plague.

## Chinese traditional patent medicines Used for COVID-19 Treatment and Relevant Clinical Studies

**FIGURE 1:**
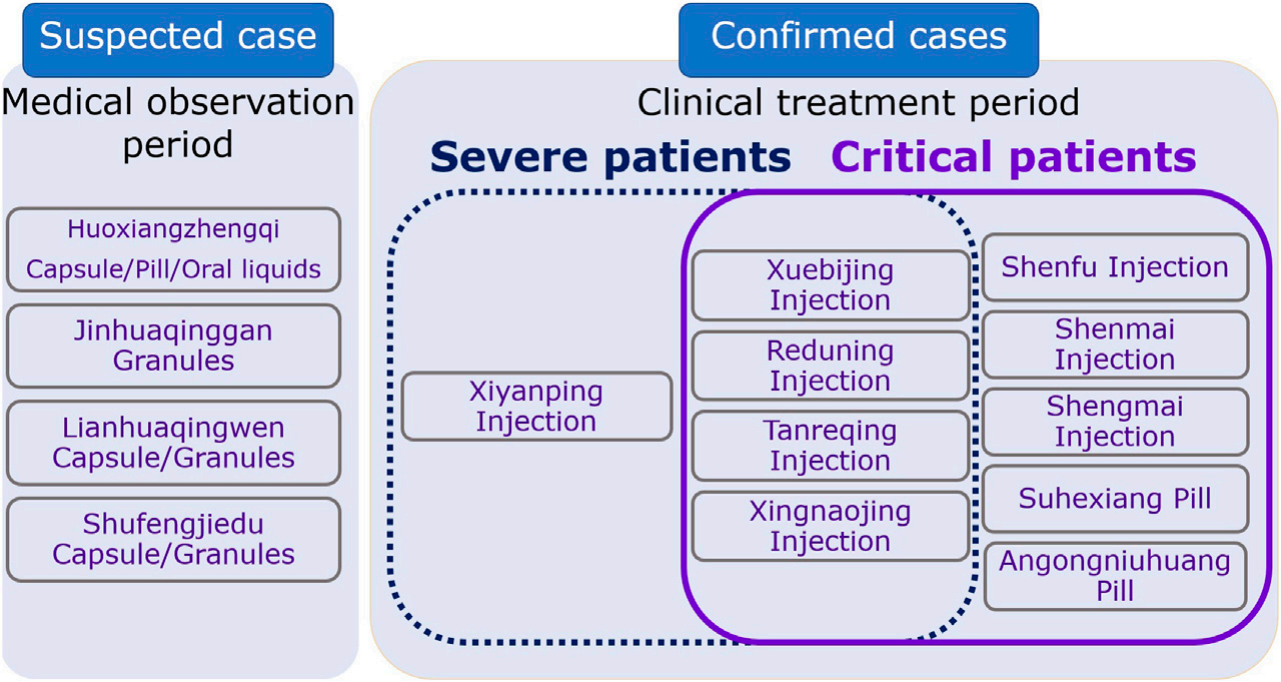
Recommended CPMs and corresponding applicable patients with COVID-19.

In < Guidelines for diagnosis and management of COVID-19 (8^th^ edition)>, 14 CPMs were recommended with six of them being of oral dosage form and eight of injection dosage form (Table 1; Table 2). For suspected COVID-19 patients or those who had close contact with these patients, medical observation was required. If they showed symptoms like fatigue, gastro-intestinal upset, or fever, 4 CPMs of oral dosage form were recommended and these included: Huoxiangzhengqi capsules for those with gastrointestinal symptoms, Jinhuaqinggan granules, Lianhuaqingwen capsules or granules, and Shufengjiedu capsules or granules for those with fever. Patients with severe symptoms were classified into two categories: Yidubifei (literal meaning: functions of the lungs were compromised due to viral infection. Patients showed symptoms of fever, face flushing, coughing with little thick yellowish sputum, or sputum with blood, shortness of breath, fatigue, dry mouth with sticky saliva, loss of appetite, difficulty in defecating, small volume of dark urine, red tongue, yellowish and greasy coating of the tongue, rapid pulsation) and Qiyingliangfan (a syndrome characterized by high fever and thirst, shortness of breath, delirium and loss of consciousness, blurred vision, maculae on the skin, hematemesis, epistaxis, seizures, red tongue with little or no coating, sunken, fine, and rapid pulsation, or superficial, large, and rapid pulsation). CPMs in the injection dosage form were recommended for these patients who were admitted due to the severe symptoms, including Xiyanping injection, Xuebijing injection, Reduning injection, Tanreqing injection, Xingnaojing injection. Shenfu injection, Shengmai injection, Shenmai injection were also recommended to use in combination with the abovementioned injection dosage forms for patients with breathing difficulty, disturbance in consciousness, and mechanical ventilation. These injections could be used alone if they are similar to each other in their therapeutic mechanism or two of them used in combination based on the condition of patients. These injections can also be used in combination with decoctions. For patients with severe symptoms, two oral dosage forms were also recommended, including Angongniuhuang and Suhexiang pills (Figure 1).

**TABLE 1:** Summary of recommended oral CPMs in the Guidelines on Diagnosis and Management of COVID-19.

Drug name	Compositions		Clinical indications
Huoxiangzhengqi Capsule/Pill/Oral liquids	Guanghuoxiang	*Pogostemon cablin* (Blanco) Benth*.*	Resolve dampness with aromatics and disperse cold as well as regulate the stomach, used for wind-cold, internal dampness stasis, or humidity induced viral infection, manifested as headache, lightheadedness, fullness of the chest, distension and pain in the belly, vomiting and diarrhea, compatible with GI type of cold.
	Zisuye	*Perilla frutescens* (L.) Britton	
	Baizhi	*Angelica dahurica* (Hoffm.) Benth. & Hook.f. ex Franch. & Sav.	
	Baizhu	*Atractylodes macrocephala* Koidz.	
	Chenpi	*Citrus × aurantium* L*.*	
	Fabanxia	*Pinellia ternata* (Thunb.) Makino	
	Houpo	*Magnolia officinalis* Rehder & E.H.Wilson	
	Fuling	*Poria cocos* (Schw. ) Wolf.	
	Jiegeng	*Platycodon grandiflorus* (Jacq.) A.DC*.*	
	Gancao	*Glycyrrhiza uralensis* Fisch. ex DC.	
	Dafupi	*Areca catechu* L*.*	
	Dazao	*Ziziphus jujuba* Mill.	
	Shengjiang	*Zingiber officinale* Roscoe	
Jinhuaqinggan Granules	Jinyinhua	*Lonicera japonica* Thunb.	Dispel the wind and ventilate the lungs, clear heat and toxin, used for fever, mild cold feeling, inflamed throat with pain, stuffy nose with nose discharge, thirst, coughing with/without sputum, red tongue, thin and yellow coat of the tongue, fast pulse due to external infection.
	Shigao	*Gypsum Fibrosum*	
	Mahuang	*Ephedra sinica* Stapf	
	Kuxingren	*Prunus armeniaca* L.	
	Huangqin	*Scutellaria baicalensis* Georgi	
	Lianqiao	*Forsythia suspensa* (Thunb.) Vahl	
	Zhebeimu	*Fritillaria thunbergii* Miq.	
	Zhimu	*Anemarrhena asphodeloides* Bunge	
	Niubangzi	*Arctium lappa* L.	
	Qinghao	*Artemisia annua* L.	
	Bohe	*Mentha canadensis* L.	
	Gancao	*Glycyrrhiza uralensis* Fisch. ex DC.	
Lianhuaqinwen Capsule/Granules	Lianqiao	*Forsythia suspensa* (Thunb.) Vahl	Clear heat and toxins, ventilatelungs and expel heat, used for syndromes of lung infection by influenza viruses, manifested as fever or high fever, cold feeling, sore muscles, stuffy nose with discharge, coughing, headache, dry and sore throat, reddish tongue, yellow or yellow thick coat of the tongue.
	Jinyinhua	*Lonicera japonica* Thunb.	
	Mahuang	*Ephedra sinica* Stapf	
	Kuxingren	*Prunus armeniaca* L.	
	Shigao	*Gypsum Fibrosum*	
	Banlangen	*Isatis tinctoria* L.	
	Mianmaguanzhong	*Dryopteris crassirhizoma* Nakai	
	Yuxingcao	*Houttuynia cordata* Thunb.	
	Guanghuoxiang	*Pogostemon cablin* (Blanco) Benth.	
	Dahuang	*Rheum officinale* Baill.	
	Hongjingtian	*Rhodiola rosea* Linn.	
	Bohenao	*Mentholum*	
	Gancao	*Glycyrrhiza uralensis* Fisch. ex DC.	
Shufengjiedu Capsule/Granules	Huzhang	*Reynoutria japonica* Houtt.	Dispel the wind and clear heat and toxins, pacify the throat, used for acute upper respiratory tract infection due to wind-heat, manifested as fever, cold feeling, sore throat, headache, stuffy nose with discharge, coughing etc.
	Lianqiao	*Forsythia suspensa* (Thunb.) Vahl	
	Banlangen	*Isatis tinctoria* L.	
	Chaihu	*Bupleurum chinense* DC.	
	Baijiangcao	*Patrinia scabiosifolia* Link	
	Mabiancao	*Verbena officinalis* L.	
	Lugeng	*Phragmites australis* (Cav.) Trin. ex Steud.	
	Gancao	*Glycyrrhiza uralensis* Fisch. ex DC.	
Angong Niuhuang Pill	Niuhuang	Calculus Bovis	Clear heat and toxins, relieve convulsion and rescue consciousness, used for high fever with seizures, delirium, coma due to stroke, encephalitis, meningitis, intracerebral hemorrhage, sepsis due to excessive heat inflicting the heart.
	Shuiniujiao	Buffalo Horn Extract	
	Shexiang	Moschus	
	Zhenzhu	Margarita	
	Zhusha	Cinnabar	
	Xionghuang	Realgar	
	Huanglian	*Coptis chinensis* Franch.	
	Huangqin	*Scutellaria baicalensis* Georgi	
	Zhizi	*Gardenia jasminoides* J.Ellis	
	Yujin	*Curcuma aromatica* Salisb.	
	Bingpian	Borneolum Syntheticum	
Suhexiang Pill	Suhexiang	*Liquidambar orientalis* Mill.	Resuscitate with aromatics, facilitate flow of Qi and relieve pain, used for coma due to phlegm blocking the heart, paresis due to stroke, heat stroke, heart pain, and stomache.
	Anxixiang	*Styrax tonkinensis* (Pierre) Craib ex Hartwich	
	Bingpian	Borneolum Syntheticum	
	Shuiniujiao	Cornu Bubali	
	Shexiang	Moschus	
	Tanxiang	*Santalum album* L*.*	
	Chenxiang	*Aquilaria sinensis* (Lour.) Spreng.	
	Dingxiang	*Syzygium aromaticum* (L.) Merr. & L.M.Perry	
	Xiangfu	*Cyperus rotundus* L.	
	Muxiang	*Aquilaria sinensis* (Lour.) Spreng.	
	Ruxiang	*Boswellia carteri* Birdw.	
	Biba	*Piper longum* L.	
	Baizhu	*Atractylodes macrocephala* Koidz.	
	Hezirou	*Terminalia chebula* Retz.	
	Zhusha	Cinnabar	

**TABLE 2:** Summary of recommended CPMs injection in the Guidelines on Diagnosis and Management of COVID-19.

Drug name	Compositions		Clinical indications
Xiyanping Injection	Andrographolide sulfonates		Clear heat and toxins, relieve coughing and diarrhea, used for bronchitis, tonsillitis, bacterial dysentery.
Xuebijing Injection	Honghua	*Carthamus tinctorius* L.	Remove statisis and clear toxins, used for warm-heat conditions, manifested as fever, panting, palpitation, dysphoria due to accumulation of toxins and stasis, applicable for systemic inflammatory reaction due to infection, and for multiple organ dysfunction at the dysfunctioning stage.
	Chishao	*Paeonia lactiflora* Pall.	
	Chuanxiong	*Conioselinum anthriscoides* ‘Chuanxiong'	
	Danshen	*Salvia miltiorrhiza* Bunge	
	Danggui	*Angelica sinensis* (Oliv.) Diels	
Reduning Injection	Qinghao	*Artemisia annua* L*.*	Clear heat and toxins, dispel the wind, used for upper respiratory infection (due to wind-cold), manifested as high fever, mild cold feeling, headache and sore muscles, coughing with yellow sputum.
	Jinyinhua	*Lonicera japonica* Thunb.	
	Zhizi	*Gardenia jasminoides* J.Ellis	
Tanreqing Injection	Huangqin	*Scutellaria baicalensis* Georgi	Clear heat and toxins, reduce phlegm, used for heat accumulating lung conditions due to wind-warm or blockage of lungs by phlegum and heatt. Manifested as fever, coughing, difficulty in clearing sputum, swelling and sore throat, thirst, red tongue with yellow coat, early stage of acute bronchitis, chronic bronchitis with acute onset, and other acute upper respiratory tract infections.
	Xiongdanfen	Bear Bile Powder	
	Shanyangjiao	Cornu Caprae	
	Jinyinhua	*Lonicera japonica* Thunb.	
	Lianqiao	*Forsythia suspensa* (Thunb.) Vahl	
Xingnaojing Injection	Shexiang	Moschus	Clear heat and toxins, cooling blood and remove stasis, restore consciousness, used for dysfunction of Qi-blood in meridians, stasis of the brain meridian leading to stroke and coma, paresis and dysarthria; headache and coma due to trauma; alcohol intoxication, headache, nausea, coma with seizures, brain embolism, acute stage of intracerebral hemorrhage, head trauma, acute toxicity of alcohol.
	Yujin	*Curcuma aromatica* Salisb.	
	Bingpian	Borneolum Syntheticum	
	Zhizi	*Gardenia jasminoides* J.Ellis	
Shenfu Injection	Hongshen	*Panax ginseng* C.A.Mey*.*	Rescue Yang and Qi, tonify Qi and prevent collapse. Used for shock due to infections, loss of blood, dehydration, and for Yang (Qi) deficiency, manifested as panic, severe palpitation, coughing and panting, stomachache, diarrhea, and paresis.
	Fupian	*Aconitum carmichaeli* Debx.	
Shenmai Injection	Hongshen	*Panax ginseng* C.A.Mey.	Tonify Qi and prevent collapse, increase Yin, Jin, and Mai. Used for shock due to Qi and Yin deficiency, coronary heart disease, viral myocarditis, chronic pulmonary heart disease, granulocytopenia, increases immunity of cancer patients during chemotherapy, reduce side effects of chemotherapy drugs.
	Maidong	*Ophiopogon japonicus* (Thunb.) Ker Gawl.	
Shengmai Injection	Hongshen	*Panax ginseng* C.A.Mey.	Tonify Qi and prevent collapse, increase Yin, used for palpitation, panting, cold limbs, perspiration, loss of pulse or myocardial infarction, cardiogenic shock, infectious shock due to Qi and Yin deficiency.
	Maidong	*Ophiopogon japonicus* (Thunb.) Ker Gawl.	
	Wuweizi	*Schisandra chinensis* (Turcz.) Baill.	

**FIGURE 2:**
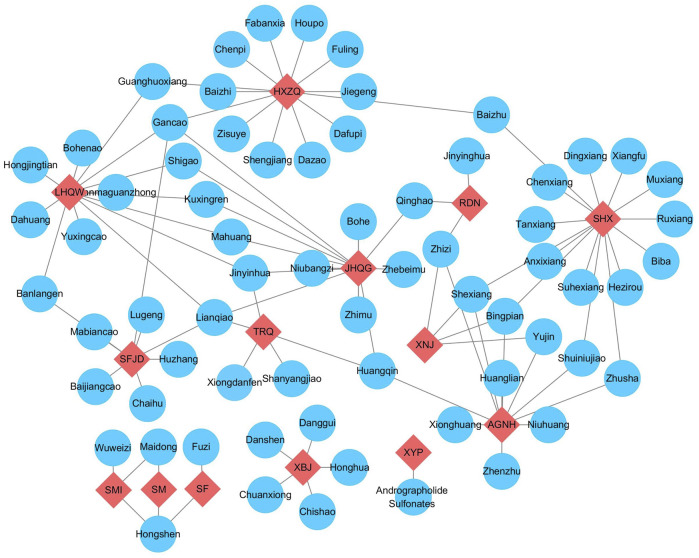
The relationship of recommended CPMs and compositions. Note: The red nodes represented different CPMs, and compositions were labelling blue. AGNH, angong Niuhuang Pill; HXZQ, huoxiangzhengqi capsule; JHQG, jinhuaqinggan granules; LHQW, lianhuaqingwen capsule; RDN, reduning injection; SF, shenfu injection; SFJD, shufengjiedu capsule; SHX, suhexiang pill; SM, shenmai injection; SMI, shengmai injection; TRQ, tanreqing injection; XBJ, xuebijing injection; XNJ, xingnaojing injection; XYP, xiyanping injection.

CPMs are generally comprised of multiple herbs. For example, Suhexiang pills are comprised of 15 herbs, but Xiyanping injection is the only one that has one effective compound. Some herbs have the same compounds. For example, Jinhuaqinggan granules, Lianhuaqingwen granules, Reduning injection, and Tanreqing injection have the same herb- *Lonicera japonica* Thunb. (Jinyinhua). Lianhuaqingwen capsules (granules), Shufengjiedu capsules, and Tanreqing injection have the same herb-*Forsythia suspensa* (Thunb.) Vahl (Lianqiao) (Figure 2).

Both Chinese and English databases were searched for relevant articles regarding the 14 recommended CPMs. These databases included China National Knowledge Infrastructure (CNKI), Wanfang database, Sinomed Database, PubMed and Embase. The time was limited to the beginning of the database to December 2, 2020. Multiple keywords for COVID-19 were used, including COVID-19, 2019 novel coronavirus, SARS-CoV-2, 2019-nCoV, coronavirus disease 2019, coronavirus disease-19. Key words for CPMs included its general names and commercial names.

By 20:00 December 2, 2020, the Chinese Clinical Trial Registry website www.chictr.org.cn/ showed 15 ongoing clinical trials on CPMs for COVID-19, including four trials on Xiyanping injection, two on Xuebijing injection, two on Reduning injection, one on Tanreqing injection, one on Shenfu injection, one on Jinhuaqing granules, and four on Lianhuaqingwen granules/capsules (Table 3).

**TABLE 3:** Clinical trials on CPMs for COVID-19.

Drug name	Registration number	Public title	Study type	Study design
Huoxiangzhengqi oral liquids	ChiCTR2000029479	Research for Traditional Chinese Medicine Technology Prevention and Control of Novel Coronavirus Pneumonia (COVID-19) in the Community Population	Interventional study	Parallel
Lianhuaqingwen Capsule/Granule	ChiCTR2000035046	Efficacy and outcomes of Lian-Hua Qing-Wen capsule in the treatment of novel coronavirus pneumonia (COVID-19): a medical records based retrospective study	Observational study	Non randomized control
	ChiCTR2000029433	A randomized, open-label, blank-controlled trial for Lian-Hua Qing-Wen Capsule/Granule in the treatment of suspected novel coronavirus pneumonia (COVID-19)	Interventional study	Parallel
	ChiCTR2000029434	A randomized, open-label, blank-controlled trial for Lian-Hua Qing-Wen Capsule/Granule in the treatment of novel coronavirus pneumonia (COVID-19)	Interventional study	Parallel
Jinhuaqinggan Granules	ChiCTR2000036871	Multi-center, Randomized, Double Blind and Placebo Controlled Clinical Trial on the Efficacy and Safety of Jinhuaqinggan Granules (JHQG) for the Treatment of COVID-19 Patients.	Interventional study	Parallel
Xiyanping Injection	ChiCTR2000030218	Study of pinavir/ ritonavir Tablets (Trade Name: Kelizhi) Combined with Xiyanping Injection for Novel Coronavirus Pneumonia (COVID-19)	Interventional study	Parallel
	ChiCTR2000032412	A medical records based retrospective study for the effectiveness and safety of Xi-Yan-Ping injection combined with conventional protocol in the treatment of common type novel coronavirus pneumonia (COVID-19)	Observational study	Non randomized control
	ChiCTR2000030117	A multicenter, randomized, open, parallel controlled trial for the evaluation of the effectiveness and safety of Xiyanping injection in the treatment of common type novel coronavirus pneumonia (COVID-19)	Interventional study	Parallel
	ChiCTR2000029756	Clinical study of nebulized Xiyanping injection in the treatment of novel coronavirus pneumonia (COVID-19)	Interventional study	Parallel
Xuebijing injection	ChiCTR2000030388	Efficacy and safety of Xue-Bi-Jing injection in the treatment of severe cases of novel coronavirus pneumonia (COVID-19)	Interventional study	Parallel
Reduning injection	ChiCTR2000029589	A prospective, randomized, open, multi-center clinical trial evaluating the efficiency and safety of Reduning injection in the treatment of novel coronavirus pneumonia (COVID-19)	Interventional study	Non randomized control
Tanreqing Injection	ChiCTR2000029432	A Real World Study For the Efficacy and Safety of Large Dose Tanreqing Injection in the Treatment of Patients with Novel Coronavirus Pneumonia (COVID-19)	Interventional study	Sequential
Tanreqing Capsules	ChiCTR2000029813	Clinical Trial for Tanreqing Capsules in the Treatment of Novel Coronavirus Pneumonia (COVID-19)	Interventional study	Parallel
Shengfu injection	ChiCTR2000030043	Shen-Fu injection in the treatment of severe novel coronavirus pneumonia (COVID-19): a multicenter, randomized, open-label, controlled trial	Interventional study	Parallel

## Chinese Traditional Patent Medicines Used in the Observation Period

Lianhuaqingwen granules are based on Maxingshigan decoction from <Shanghanlun (Treatise on febrile caused by cold) > and Yinqiaosan from <Wenbingtiaobian (Differentiation on Febrile Diseases)>. The major therapeutic effects include opening the lungs as well as clearing the heat and toxins (Li H. R. et al., 2020). Based on findings from network pharmacology and molecular docking analyses, Lianhuaqingwen capsules fight against COVID-19 through multi-components, multi-targets, and multi-pathways. The major effective compounds of this herbal product bind to the main protease (Mpro) and angiotensin converting enzyme II (ACE2) of SARS-CoV-2 with high affinity, through which COVID-19 is inhibited (Ling et al., 2020; Xia et al., 2020). A pharmacodynamic study showed that Lianhuaqingwen granules significantly inhibited the replication of COVID-19 and transformed the morphology of this virus *in vitro* (Li et al., 2020b). Another study showed that Lianhuaqingwen capsules inhibited the replication of multiple influenza viruses *in vitro* and the 50% inhibitory concentration (IC50) of Lianhuaqingwen capsules was in the range of 0.35 mg/ml∼2 mg/ml (Ding et al., 2017). A prospective, multi-centered, randomized controlled clinical study investigated the recovery rate of main symptoms including fever, fatigue, and coughing by dividing 284 COVID-19 patients from 23 hospitals of nine provinces in China into the conventional treatment group and the Lianhuaqingwen granule group. It was found that the Lianhuaqingwen group had a cure rate of 91.5%, which was significantly higher than that of the conventional group-82.4% (independent *t* test, *p* = 0.022). The median time of recovery was significantly shorter in the Lianhuaqingwen group than in the conventional group (7 vs 10 days, *p* < 0.001). This was also observed in the duration of fever (2 vs 3 days, *p* < 0.001), fatigue (3 vs 6 days, *p* < 0.001), and coughing (7 vs 10 days, *p* < 0.001). 83.8% of patients in the Lianhuaqingwen group showed improvement on chest computed tomography compared to 64.1% in the conventional group (*p* < 0.001), and 78.9% of patients were cured in the Lianhuaqingwen group compared to 66.2% in the conventional group (*p* = 0.017). However, no difference was found in the proportion of patients progressed into the critical stage or in the viral test (*p* > 0.05) (Hu et al., 2020). Another study examined 151 severe patients with confirmed COVID-19 on their coagulation, white cell count, and prognosis after 25 days of treatment with ribavirin, ritonavir, umifenovel, and Lianhuaqingwen. It was found that coagulation and white cell count were significantly improved after the treatment and their prognosis was good. Therefore, it was recommended to be the first line treatment for this type of patients (Li, 2020). In a study on suspicious COVID-19 patients, the proportions of patients without fever, coughing, and malaise were 86.7, 55.6, and 82.5% in the Lianhuaqingwen group with conventional treatment and Lianhuaqingwen after receiving the treatment compared with 67.7, 30.6, and 58.6% in the conventional treatment group (all *p* < 0.05). Similar results were found in the disappearance of shortness of breath and moist rales between the two groups (68.2 vs 20.0%, 56.0 vs 20.0%, *p* < 0.05). No significant difference was found in the duration of fever and ratios of patients progressed to the critical stage (Lv et al., 2020). In a retrospective study on 214 mild/general COVID-19 patients who were treated with arbidol or in combination with Lianhuaqingwen capsules, it was found that patients treated with arbidol and symptomatic therapy had a higher incidence of progressing to the critical stage than those treated with both arbidol, symptomatic therapy, and Lianhuaqingwen (*χ* ^*2*^ = 16.823, *p* = 0.001). There was no significant difference in the duration of admission (*χ* ^*2*^ = 2.889, *p* = 0.409) (Yu et al., 2020). Another study reported positive results on fever, coughing, malaise, and other symptoms after treatment with Lianhuaqingwen, but there was no control group in that study and the level of evidence is low (Cheng and Li, 2020).

Huoxiangzhengqi pills were first recorded in <Taipinghuiminhejijufang (Formularies of the Bureau of People’s Welfare Pharmacies)>. This herbal recipe is known for resolving dampness with aromatics and dispersing cold as well as regulating the stomach. Modern pharmacological research has shown that this herbal product has anti-spasm, analgesic, anti-bacterial, anti-viral, anti-emetic, and regulating functions of the stomach and the intestines (Huo et al., 2020). Network pharmacology and molecule docking techniques revealed that compounds in Huoxiangzhengqi oral liquid took its anti-viral effect by modulating multiple pathways, such as prostaglandin-endoperoxide synthase (PTGS-2), Heat Shock Protein 90 Alpha Family Class B Member 1 (HSP90AB1), and calmodulin-regulated spectrin-associated protein 2 (CAMSAP2), after binding to ACE2 (Deng et al., 2020). In a prospective, randomized, parallel controlled preventive study, Huoxiangzhengqi oral liquid was prescribed in combination with Jingaojiere granules to COVID-19 patients. A total of 22,065 recipients were divided into two groups: the health briefing group and the herbal group. The latter group was also briefed with hygiene measures. During the 14 days follow up period, neither of the groups had COVID-19 patients, but the herbal group had fewer participants suffering from the cold than the health briefing group (*p* < 0.05). Herbal products protected 91.8% of the population, especially people aged 16–60 years (Yan et al., 2020). The other study showed that Huoxiangzhengqi pills along with Lianhuaqingwen granules decreased the use of macrolides by COVID-19 patients, but did not show superiority in disease deterioration than conventional treatment. However, combined herbal therapy did decrease the proportion of patients progressed to the critical stage, demonstrating the potential of combined use of herbal products and conventional therapies in improving the outcomes of COVID-19 patients (Xiao et al., 2020).

Jinhuaqinggan granules derived from Yinqiaosan and Maxingshigantang by removing some herbs and adding others. This herbal product is known for dispelling the wind, ventilating the lungs, clearing the heat and toxins (Mao et al., 2020). Network pharmacology research found that it had multiple effective compounds, like formononetin, stigmasterol, β-quebrachol, icaritin, etc., which regulated multiple pathways, such as PTGS2, HSP90AB1, HSP90AA1, PTGS1, through binding to SARS-CoV-2 3C-like protease (3CL^pro^) and ACE2, resulting in the control of COVID-19 (Gong et al., 2020). In another study, 123 COVID-19 patients were randomly allocated to the combined treatment group (Jinhuaqinggan granules and conventional treatment) and the conventional treatment group in a ratio of 2:1. After 5 days treatment, the combined treatment group showed significant improvement in fever (80.3 vs 53.1%, *p* < 0.05), coughing (66.1 vs 42.9%, *p* < 0.05), malaise (77.6 vs 53.8%, *p* < 0.05), expectoration (85.3 vs 46.2%, *p* < 0.05). Scores in the TCM syndrome scale and Hamilton depression scale were significantly lower in the combined treatment group than in the conventional group (*p* < 0.01) (Duan et al., 2020). In a retrospective study, 80 COVID-19 patients were divided into two groups, receiving Jinhuaqinggan granules and conventional treatment, respectively. It was found that the duration of positive virus detection was shorter in the Jinhuaqinggan group than in the conventional group (7 ± 4 days vs 10 ± 4 days, *p* < 0.05). The virus clearance rate was higher (56.82 vs 27.78%, *p* < 0.05) and the time to recover as shown on chest computed tomography (CT) was shorter (8 ± 4 days vs 10 ± 5 days, *p* = 0.021) in the Jinhuaqinggan group than in the conventional group, respectively (Liu et al., 2020).

Shufengjiedu capsules are one of the essential herbal recipes in managing acute upper respiratory tract infection. Animal experiments, network pharmacology, as well as genetic studies showed that this herbal product had multi-component, multi-target, and multi-pathway characters (Zhang et al., 2016). In the acute lung injury model, Shufengjiedu capsules were able to suppress inflammation induced by LPS (lipopolysaccharide) and to relieve lung injuries due to endotoxins, which might be mediated by inhibition of the mitogen-activated protein kinases (MAPK)/nuclear factor kappa B (NF-κB) signaling pathway and downregulation of NF-κB mRNA (Tao et al., 2014). Network pharmacology and molecule docking techniques revealed that compounds, extracted from this herbal product including quercetin, luteolin, kaempferol, wogonin and acacetin, were able to regulate multiple pathways through binding to key target proteins like interleukin 6 (IL-6), albumin (ALB), MAPK3, and others, resulting in the control of COVID-19 (Shen et al., 2020). In a retrospective study on 200 general COVID-19 patients who were treated with arbidol hydrochloride or in combination with Shufengjiedu capsules, it was found that patients treated with arbidol hydrochloride and Shufengjiedu capsules had a shorter time in defervescence, larger white blood cell counts, higher percentages of lymphocytes and lower serum C-reactive protein (CRP) as well as IL-6 than those treated with arbidol hydrochloride alone, respectively. As expected, the combined treatment group had a higher percentage of clear chest CT than the arbidol hydrochloride group (Chen et al., 2020a).

## Herbal Products for Severe or Critical COVID-19 Patients

Xiyanping injection is a single compound formula obtained after sulfonating Andrographolide extracted from *Andrographis paniculate*. It has non-specific, anti-inflammatory, anti-viral effect and has been recommended to treat H1N1 influenza, epidemic colds (influenza B), H7N9 avian influenza, community acquired pneumonia (Wang ZF. et al., 2019). To investigate the anti-inflammatory effect of Andrographolide, mRNA levels of inflammatory biomarkers, such as toll-like receptor 4 (TLR4), CD14 and myeloid differentiation factor 2 (MD2), were examined in a rat model of acute lung injury. It was found that Andrographolide decreased mRNA levels of TLR4, CD14 and MD2 in LPS induced acute lung injury (Xu et al., 2015), suppressed inflammation mediated by NF-κB and MAPK (Peng et al., 2016), and alleviated pathologies of the lungs. A meta-analysis study on community acquired pneumonia showed that Reduning injection, Yanhuning injection, Xiyanping injection, and Tanreqing injection, when used in combination with conventional treatment, showed better therapeutic effect than conventional treatment alone. Tanreqing plus conventional treatment showed the best result in reducing the duration of fever and the average length of stay. Xiyanping plus conventional treatment showed the best result in reducing moist rales in the lungs (Li et al., 2017). In a prospective, randomized controlled study on hand-foot-and-mouth disease, 451 severe pediatric patients were divided into the Xiyanping or Reduning plus conventional treatment group and the conventional treatment group. It was found that the conventional treatment group had a longer half time of defervescence (40.4 vs 27.2 h, *p* < 0.01), a lower clearance of rashes (43.6 vs 29.5%, *p* < 0.01), higher proportions of patients without coughing, malaise, fragility, agitation or irritability (32.6 vs 19.2%, *p* < 0.01) than the combined group, respectively. Xiyanping had similar therapeutic effect on mild hand-foot and mouth disease (Zhang G. et al., 2017). A meta-analysis of the therapeutic effect of Xiyanping plus azithromycin on Chinese pediatric pneumonitis due to mycoplasm infection included nine trials and 963 patients. It showed that Xiyanping plus azithromycin were superior in duration of admission, time to defervescence, time to stop coughing, time to disappearance of moist rales than azithromycin alone (Li Q. et al., 2019).

Xuebijing injection is comprised of extracts from *Carthamus tinctorius* L*.* (Honghua), *Paeonia lactiflora* Pall*.* (Chishao), C*onioselinum anthriscoides* “Chuanxiong” (Chuanxiong), *Salvia miltiorrhiza* Bunge (Danshen), *Angelica sinensis* (Oliv.) Diels (Danggui). It promotes blood circulation to remove blood stasis and clears toxins, for which it has been used to treat systemic inflammatory response syndrome and multiple organ dysfunction at the dysfunctioning stage (Shi et al., 2020). Its therapeutic effect was recognized by clinical experts (Li C. et al., 2018; Song et al., 2018). Network pharmacology research showed that multiple compounds shared a number of targets with COVID-19. Among them, IL6, tumor necrosis factor alpha (TNFα), MAPK1, MAPK14, MAPK8, PTGS2, IL2 and PPARG were the predominant targets for Xuebijing to treat COVID-19. The major effective compounds of this herbal product, including luteolin, quercetin, kaempferol, β-sitosterol, stigmasterol, were able to bind to 2019-nCoV 3CLpro and ACE2 to resist COVID-19 (Huang et al., 2020). In a study on Xuebijing injection, 60 severe COVID-19 patients were divided into conventional treatment group, 50 ml Xuebijing group, and 100 ml Xuebijing group on a 1:1:1 basis. After 8 days treatment, the Acute physiology and chronic health evaluation Ⅱ (APACHE-Ⅱ) score was lower in the 100 ml Xuebijing group than in the conventional treatment group and the 50 ml Xuebijing group [APACHE-Ⅱ score: 12.3 ± 1.5 (100 ml Xuebijing group) vs 16.5 ± 1.6 (conventional treatment group) vs 15.9 ± 1.4 (50 ml Xuebijing group), *p* < 0.05, respectively). After treatment, the COVID-19 nucleic acids of some patients turned negative, but there was no significant difference between groups. The 100 ml Xuebijing group showed significantly greater improvement than the 50 ml Xuebijing group (*p* < 0.05) and the conventional treatment group (*p* < 0.05) (Wen et al., 2020). In another study, 42 COVID-19 patients received Xuebijing plus conventional treatment (including electrolytes, blood glucose and blood pressure management, nutritional support, oxygen therapy and antiviral treatment) and patients in the control group received conventional treatment alone. After 7 days treatment, the combined group showed significant improvement in IL-6 and temperature than the conventional group with a larger reduction in temperature in the combined group (*p* < 0.05), especially in severe patients. The proportion of patients with improvement in chest CT and COVID-19 nucleic acids turning negative was higher in the combined group than in the conventional group, but this difference was not statistically significant (*p* > 0.05) (Guo et al., 2020). In another study, 60 patients with COVID-19 were divided into the conventional treatment group and the Xuebijing group (conventional treatment plus Xuebijing). It was found that Xuebijing significantly suppressed IL-6, IL-8 and TNF-α (*p* < 0.05). There was no significant difference in adverse events such as allergic shock (Luo et al., 2021). In a prospective, randomized controlled study on 710 patients with community acquired pneumonitis, it was shown that Xuebijing significantly decreased the mortality rate on day 28 (15.87 vs 24.63%, *p* = 0.006), the time in need of mechanical ventilation (11 vs 16.5 days, *p* = 0.012), and duration of admission (12 vs 16 days, *p* = 0.004) than placebo, respectively. There was no significant difference in adverse events between the two groups (Jiang et al., 2019; Song et al., 2019).

Reduning injection is comprised of extracts from *Artemisia annua* L*.* (Qinghao), *Lonicera japonica* Thunb*.* (Jinyinhua), *Gardenia jasminoides* J.Ellis (Zhizi). It has been widely used for upper respiratory tract infection due to its superiority in clearing the heat, dispelling the wind, and clearing toxins. Its therapeutic effect on COVID-19 might be attributed to its anti-inflammatory and multi-target, multi-pathway immune modulating characters (Jiang et al., 2019). Interacting proteins of SARS-CoV-2 and humans have been mapped, among which human proteins like ATP binding cassette C1 (ABCC1), sigma receptor 1 (SigmaR1), and inosine monophosphate dehydrogenase type Ⅱ (IMPDH2) were targeted by Reduning to interrupt interactions between viral and human proteins. As a result, the invasion process of COVID-19 was discontinued. As a result, infection by COVID-19 was prevented (Chen S. J. et al., 2020). In a multi-centred, randomized clinical trial, the therapeutic effect and safety of Reduning were investigated in 12 hospitals in China. Patients receiving Reduning injections and conventional treatment had a higher percentage of mitigated symptoms after 14 days treatment compared with those receiving conventional treatment alone [full-analysis set (FAS): 84.4 vs 60.0%, *p* = 0.0004]. They also had shorter median time to symptom alleviation (143 h *vs* 313.5 h, *p* < 0.001), time to nucleic acid turning negative (146.5 h *vs* 255.5 h, *p* < 0.001), length of stay (4.1 d *vs* 18.1 d, *p* < 0.001), and time to defervescence (29 h *vs* 71 h, *p* < 0.001) than the control group. No significant difference in adverse events were found between these two groups (3.9% *vs* 8.8%, *p* = 0.383) (Xu et al., 2021). In a previous multi-centred, stratified randomized, double blind, parallel controlled study on hand-foot and mouse disease, patients were randomly divided into three groups with 120 in each group. The first group received conventional treatment, the second group received Reduning injection, and the third group received Reduning plus conventional treatment. It was found that the combined group was more superior in defervescence than the conventional group, and more superior in managing rashes than the conventional group and the Reduning group. But there was no significant difference in managing stomatological ulcer between these three groups (Zhang et al., 2013). A meta-analysis on Reduning managing acute tracheobronchitis showed that Reduning increased the effectiveness rate, decreased the duration of fever, coughing, rales in the lungs, and the incidence of adverse events which were minor in nature. However, the quality of this study was low, and its conclusion should be verified in larger-sampled, well designed, standardized clinical trials (Dang et al., 2019).

Tanreqing injection is the modified form of Shuanghuanglian by adding two heat clearing herbs-cornu gorais and bear gall powder. It clears heat, resolves phlegm, and clears toxins, for which it has been used to treat wind-warm lung heat disease (main symptoms include fever, coughing, chest pain) and lung diseases due to phlegm and heat blocking. The major compounds include baicalin, wogonoside, wogonin, ursodeoxycholic acid, chenodeoxycholic acid, chlorogenic acid, neochlorogenic acid, cryptochlorogenic acid, forsythiaside and others (Li C. et al., 2019). The therapeutic mechanism might be related to the high affinity of its major effective compounds like kaempferol, quercetin, baicalein, luteolin, and wogonin with SARS-CoV-2 3CL^pro^ (Kong et al., 2020). Previous studies showed that Tanreqing has been prescribed with antibiotics to increase the bactericidal effect and to decrease the resistance of bacteria to antibiotics (Chang, 2017; Tang et al., 2019). When used with vancomycin or linezolid at concentrations lower than their minimal inhibitory concentrations for methicillin-resistant *Staphylococcus aureus in vitro*, the synergistic bactericidal effect was stronger than each antibiotic when used alone (Yang et al., 2018). There is only one study which has reported the therapeutic effect of Tanreqing capsules on COVID-19. In this retrospective cohort study on 82 mild and moderate COVID-19 patients, it was found that the time to negative COVID-19 DNA in the stool (4 days vs 9 days, *p* = 0.047) and the duration of throat-stool negative COVID-19 DNA (0 vs. 2 days, *p* = 0.042) were shorter in the Tanreqing group than in the control group. These might be related to the increase in CD3^+^T cells (Zhang et al., 2020a). In a meta-analysis on acute onset of chronic bronchitis, 23 randomized controlled trials (RCTs) were included with 1,901 participants. It was found that time to defervescence was significantly shorter in the Tanreqing plus conventional treatment group than in the conventional treatment group. Similar superiority was also observed in the total effective rate. When used along with levofloxacin, cefuroxime, cefperazone- sulbactam, Tanreqing showed synergy with these antibiotics. Though this superiority was observed in this meta-analysis, the conclusion should be verified in future large-scaled, high-quality studies due to the small sample sizes of included studies and the low quality of study design (Gao et al., 2019).

Xingnaojing injection is derived from the classical herbal decoction Angongniuhuangwan after refining the major components of this recipe using modern pharmacological techniques. It clears the heat and toxins, cools and invigorates the blood, and revitalize the brain. It has been widely used for intracerebral hemorrhage, brain ischemia, and other neurological diseases (Zhang S. et al., 2017). *In vitro* experiments showed that the therapeutic effect on loss of consciousness is due to its anti-neurotoxicity induced by excitatory amino acids, radical scavenging, cytokine suppressing, and apoptosis related gene regulating characters (Ma et al., 2018). It can be used for COVID-19 patients with high fever and disturbance in consciousness, but there is no such report. In a meta-analysis on Xingnaojing managing coma due to high fever, stroke, and intoxication, it was found that Xingnaojing was superior in improving consciousness for such patients (Wu et al., 2016). An ongoing clinical trial- Xingnaojing for moderate-to-severe acute ischemic stroke (Lai *et al.*) aimed to investigate whether Xingnaojing is able to improve outcomes of ischemic stroke patients at 3 months after stroke onset by running a multi-centred, prospective, randomized controlled, open label trial. The result is still pending (Lai et al., 2017).

Angongniuhuang pills and Suhexiang pills can be used for resuscitation of severe or critical COVID-19 patients. Both of them are of rapid effect and potent in revitalizing the brain and are used for resuscitation of patients due to diverse critical conditions (Bian and Zhang, 2016; Liang et al., 2019). Based on the fact that the majority of patients with critical conditions have loss of consciousness and accompanying dysphagia, there is an increased risk of aspiration and choking when taking oral dosage forms. Therefore, these herbal products can be dissolved in water and dosed through the naso-gastric tube to the stomach of patients. Both of them are used for critical patients with internal closure and external detachment. However, Angongniuhuang pills are for internal closure with heat, and Suhexiang pills for internal closure with cold (Wang et al., 2020). Currently, there is no report on their therapeutic effect on severe COVID-19. In a meta-analysis on Angongniuhuang pills for stroke, 18 clinical trials including 1,601 participants were studied. It was found that Angongniuhuang pills significantly improved outcomes of ischemic stroke patients [relative risk (RR): 1.27; 95% confidence interval (Health Emergency Committee of The Chinese Research Hospital Association, 2019): 1.14–1.41] and ICH patients (RR: 1.26; 95%CI:1.14–1.38), decreased the neurological deficit scores of ischemic stroke patients [weighted mean difference (WMD) −3.52; 95% CI: −5.51 to −1.54] and ICH patients (WMD −3.64; 95% CI: −4.97 to − 2.31), and improved the Glasgow Coma scores of such patients (WMD 1.18; 95% CI: 0.79–1.56; WMD 2.28; 95% CI: 1.37–3.19) (Liu et al., 2019).

In addition, another three injections containing ginseng have been used for patients with collapse syndrome. They are Shenfu injection, Shenmai injection, and Shengmai injection (Li et al., 2016; Cao et al., 2019). Shengmai injection is derived from Shengmaisan and Shenmai injection from Maidongyin, the latter has one herb—*Schisandra chinensis* (Turcz.) Baill. (Wuweizi) less than the former one. Due to the similarity to each other, they are of similar effect on certain diseases. Deficiency in Yang or Yin should be differentiated before determining which to prescribe. Patients with Yang deficiency can be prescribed Shenfu injection, those with Yin deficiency Shengmai or Shenmai injection. In a meta-analysis including 30 RCTs and 2,038 participants, it was found that Shenmai injection plus conventional treatment was more superior in managing patients with shock (septic shock, cardiogenic shock, hypovolemic shock, neurogenic shock, allergic shock) than conventional treatment (Li X. et al., 2018). In another meta-analysis including 23 RCTs and 1,804 participants, Shengmai injection plus conventional treatment significantly improved pulmonary function, blood-gas index, immunoglobins, and time to disappearance of rales of patients with chronic obstructive pulmonary diseases (COPD). It also significantly decreased the COPD Assessment Test (CAT) score, modified Medical Research Council (mMRC), and the average duration of admission. Currently, little evidence is available for their use on COVID-19 (Huang et al., 2019). Further pharmacological research and clinical trials are needed to confirm their efficacy in managing COVID-19.

## Adverse Events and Other Notes

CPMs are made of natural herbs processed using traditional techniques. Their clinical efficacy and adverse events are not well known, which misleads the public to believe that herbs are safe and non-toxic. As adverse events of drugs are closely surveilled, more and more studies are available to report the potential toxicity and adverse events of herbs. Compared with other forms of herbal products, injections are more likely to result in adverse events, especially severe adverse events, such as allergic shock. Previous studies have shown that over 70% of adverse events of Xuebijing (Guo et al., 2017), Reduning (Sun et al., 2019), Shengmai (Cheng, 2011), Shenmai (Xiang et al., 2017), Xiyanping (Chen et al., 2018) occurred in the first half an hour. A large-scale, multi-centred Chinese study reported the incidences of adverse events and adverse reactions of Tanreqing, which were 1.4 and 0.3%, respectively. The most common adverse reaction was changes in the skin or the subcutaneous tissue. The majority (72.8%) of these reactions occurred in the first 2 hours after Tanreqing injection. Among these patients, two thirds of them can be treated with externally used drugs. Increased adverse reactions were related to history of food allergy [odds ratio (Lai *et al.*) 4.50, 95% CI: 1.35–15.00], drug allergy (OR 2.77, 95% CI: 1.56–4.94), and rapid infusion of these products (OR 2.10, 95% CI: 1.27–3.50) (Li XX. et al., 2019). A meta-analysis on Xuebijing showed that it has an incidence of adverse reactions of 5.62%, allergy of 3.16%, and the incidence of allergy increased as dosages and age increased (Wang C. et al., 2019). A meta-analysis study showed that Xiyanping injection, when used in combination with antibiotics to treat community acquired pneumonia, elicited similar adverse reactions to antibiotics alone. These reactions included gastrointestinal discomfort, dizziness, rashes, and phlebitis (Wang et al., 2018).

Certain herbs have toxic compounds which may lead to adverse reactions. For example, Suhexiang pills and Angongniuhuang pills have cinnabar (HgS) and realgar (As_2_S_2_), which cannot be used for a long term (Zhao et al., 2015). When heated, both cinnabar and realgar showed increased toxicity. Hence, they cannot be boiled, instead they can be mixed with warm water before use (Zhang et al., 2020b). Huoxiangzhengqi oral liquid has ethanol inside, which reacts with metronidazole, furazolidone, tolbutamide, and some cephalosporins with a methiazolium side chain, leading to disulfiram-like reactions which are similar to drunken behaviors (Liu and Xie, 2017). In addition, there is a lack of similar studies on certain populations, like pregnant women, lactating women, children, elderlies, and those with liver and kidney dysfunction. Therefore, caution should be taken when using herbal products. More challenges will be encountered in the prevention of COVID-19 using TCM methods, which require pharmacologists or pharmacists to provide more precise and complete service to clinicians. This will guarantee the safe prescription and efficacy of herbal products in fighting against COVID-19. In the meantime, correct clinical diagnosis is essential in managing COVID-19 patients, which will facilitate the correct use of both herbal products and western medicines with an aim to minimize the side effects and maximize the therapeutic effect. This will reflect the importance of TCM in fighting against COVID-19.

## Conclusion

Current clinical studies on COVID-19 treated with TCM have certain limitations, such as small numbers of trials, small sample sizes, low quality of methodologies, and others. Large-scaled, randomized controlled, real-world studies are still lacking, especially as adjuvant therapies for critical patients (Suhexiang pills, Angongniuhuang pills, Shenfu injection, Shengmai injection, Shenmai injection). The present study only reviewed 14 herbal products recommended by the < Guidelines for diagnosis and management of COVID-19 (8^th^ edition)>. In fact, there are many more herbal products that might be effective in treating COVID-19 (Su et al., 2020; Ye et al., 2020; Zhao et al., 2020). As convenient, highly effective, and modern TCM products, herbal products have shown excellent clinical efficacy and tremendous potential in COVID-19 prevention and treatment. It is likely to become an alternative in fighting against this pandemic for both suspected patients or populations at a high risk.

## Abbreviations

ACE2, angiotensin converting enzyme II; 3CLpro, 3C-like protease; CAMSAP, calmodulin-regulated spectrin-associated protein; CI, confidence interval; COPD, chronic obstructive pulmonary diseases; CPM, Chinese patent medicine; CT, computed tomography; HSP, heat shock protein; IC, inhibitory concentration; ICH, intracerebral hemorrhage; IL, interleukin; LPS, Lipopolysaccharide; MAPK, mitogen-activated protein kinases; NF-Κb, nuclear factor kappa B; OR, odds ratio; PTGS, prostaglandin-endoperoxide synthase; RCT, randomized controlled trial; RR, relative risk; SARS-CoV-2, severe acute respiratory syndrome coronavirus 2; TCM, traditional chinese medicine; TNF, tumor necrosis factor alpha; WMD, weighted mean difference.
